# Comparison of the Impact of Wikipedia, UpToDate, and a Digital Textbook on Short-Term Knowledge Acquisition Among Medical Students: Randomized Controlled Trial of Three Web-Based Resources

**DOI:** 10.2196/mededu.8188

**Published:** 2017-10-31

**Authors:** Michael A Scaffidi, Rishad Khan, Christopher Wang, Daniela Keren, Cindy Tsui, Ankit Garg, Simarjeet Brar, Kamesha Valoo, Michael Bonert, Jacob F de Wolff, James Heilman, Samir C Grover

**Affiliations:** ^1^ Division of Gastroenterology St. Michael's Hospital University of Toronto Toronto, ON Canada; ^2^ Department of Pathology and Molecular Medicine McMaster University Hamilton, ON Canada; ^3^ Acute Medicine Northwick Park Hospital London United Kingdom; ^4^ Department of Emergency Medicine University of British Columbia Cranbrook, BC Canada

**Keywords:** medical education, medical students

## Abstract

**Background:**

Web-based resources are commonly used by medical students to supplement curricular material. Three commonly used resources are UpToDate (Wolters Kluwer Inc), digital textbooks, and Wikipedia; there are concerns, however, regarding Wikipedia’s reliability and accuracy.

**Objective:**

The aim of this study was to evaluate the impact of Wikipedia use on medical students’ short-term knowledge acquisition compared with UpToDate and a digital textbook.

**Methods:**

This was a prospective, nonblinded, three-arm randomized trial. The study was conducted from April 2014 to December 2016. Preclerkship medical students were recruited from four Canadian medical schools. Convenience sampling was used to recruit participants through word of mouth, social media, and email. Participants must have been enrolled in their first or second year of medical school at a Canadian medical school. After recruitment, participants were randomized to one of the three Web-based resources: Wikipedia, UpToDate, or a digital textbook. During testing, participants first completed a multiple-choice questionnaire (MCQ) of 25 questions emulating a Canadian medical licensing examination. During the MCQ, participants took notes on topics to research. Then, participants researched topics and took written notes using their assigned resource. They completed the same MCQ again while referencing their notes. Participants also rated the importance and availability of five factors pertinent to Web-based resources. The primary outcome measure was knowledge acquisition as measured by posttest scores. The secondary outcome measures were participants’ perceptions of importance and availability of each resource factor.

**Results:**

A total of 116 medical students were recruited. Analysis of variance of the MCQ scores demonstrated a significant interaction between time and group effects (*P*<.001, η_g_^2^=0.03), with the Wikipedia group scoring higher on the MCQ posttest compared with the textbook group (*P*<.001, *d*=0.86). Access to hyperlinks, search functions, and open-source editing were rated significantly higher by the Wikipedia group compared with the textbook group (*P*<.001). Additionally, the Wikipedia group rated open access editing significantly higher than the UpToDate group; expert editing and references were rated significantly higher by the UpToDate group compared with the Wikipedia group (*P*<.001).

**Conclusions:**

Medical students who used Wikipedia had superior short-term knowledge acquisition compared with those who used a digital textbook. Additionally, the Wikipedia group trended toward better posttest performance compared with the UpToDate group, though this difference was not significant. There were no significant differences between the UpToDate group and the digital textbook group. This study challenges the view that Wikipedia should be discouraged among medical students, instead suggesting a potential role in medical education.

## Introduction

Health care professionals and trainees are challenged to keep pace with a rapidly growing knowledge base. By 2020, the estimated doubling time of medical knowledge will be 73 days [1]. Ubiquitous Internet accessibility has both mediated this rapid dissemination of research and allowed for increased access to information [2]. In particular, many medical trainees use Web-based resources to answer clinical questions and acquire medical knowledge [3-5]. Despite widespread use, there is a paucity of research on the impact of these resources on knowledge acquisition in medical education.

Among medical students, three commonly used Web-based resources are digital textbooks; UpToDate, a point-of-care online medical software; and Wikipedia, a freely editable encyclopedia. Previous studies have reported that a majority of medical students use UpToDate for clinical activities such as patient admissions and teaching rounds [6,7]. Textbooks, meanwhile, are more commonly used for preparation of tutorials and tests, as well as for in-depth reading [7,8]. Finally, up to 94% of medical students and 70% of junior physicians have reported using Wikipedia to supplement curricular learning and clinical practice, respectively [9,10].

Although commonly used, trainees are actively discouraged from using Wikipedia as an information source [11]. Critics argue that it is error-prone because of a lack of traditional editorial controls [12]. Moreover, studies of Wikipedia entries in cardiovascular sciences, gastroenterology, and pharmacology have found inaccuracies because of errors and omissions [11,13-15]. This skepticism, however, may be exaggerated [16-18]. A 2005 *Nature* investigation found similar error rates when comparing Wikipedia articles with their counterparts in the Encyclopedia Britannica, a trusted, expert-reviewed resource [19]. In addition, articles in gastroenterology and nephrology were shown to have moderate to fair reliability for patients [16,17]. Whereas previous reports have looked largely at the quality of Wikipedia content, there is no reported data on the direct impact of Wikipedia on knowledge acquisition.

The aim of this study was to evaluate the impact of Wikipedia on short-term knowledge acquisition among medical students compared with UpToDate and a digital textbook.

## Methods

This parallel-arm, randomized controlled trial (RCT) was conducted from April 2014 to December 2016. Approval was granted by the University of Toronto Research Ethics Board (Protocol Reference # 30420). Written consent was obtained online from all participants. All authors reviewed and approved the final manuscript. No changes to methodology after trial commencement were made. This trial was not registered as it does not meet the International Committee of Medical Journal Editors’ criteria for the definition of a clinical trial. Specifically, the outcomes tested are not patient-related outcomes.

### Participants

Preclerkship medical students were recruited from four Canadian medical schools over 2 years from April 2014 to December 2016. Convenience sampling was used with informal recruitment through word of mouth, social media, and email by 2 authors (RK and DK). The primary inclusion criterion was that participants must be medical students in preclerkship training (ie, in their first or second year of medical school) at a Canadian medical school. After recruitment, participants were anonymized with a unique identifier and randomized in an allocation ratio of 1:1:1 to one of the three groups: (1) Wikipedia, (2) UpToDate, and (3) digital textbook. The random allocation sequence was created by one author (MAS) using a Web-based random number generator. Another author (CW) assigned participants to each of the three groups using sequentially numbered assignments. Participants were blinded to group assignment until they were required to use their intervention. Once they began using their Web-based resource, blinding was not possible. Data analysts were blinded to group assignment. Participants were not told which Web-based resource was the intervention of interest.

### Study Design

**Figure 1:**
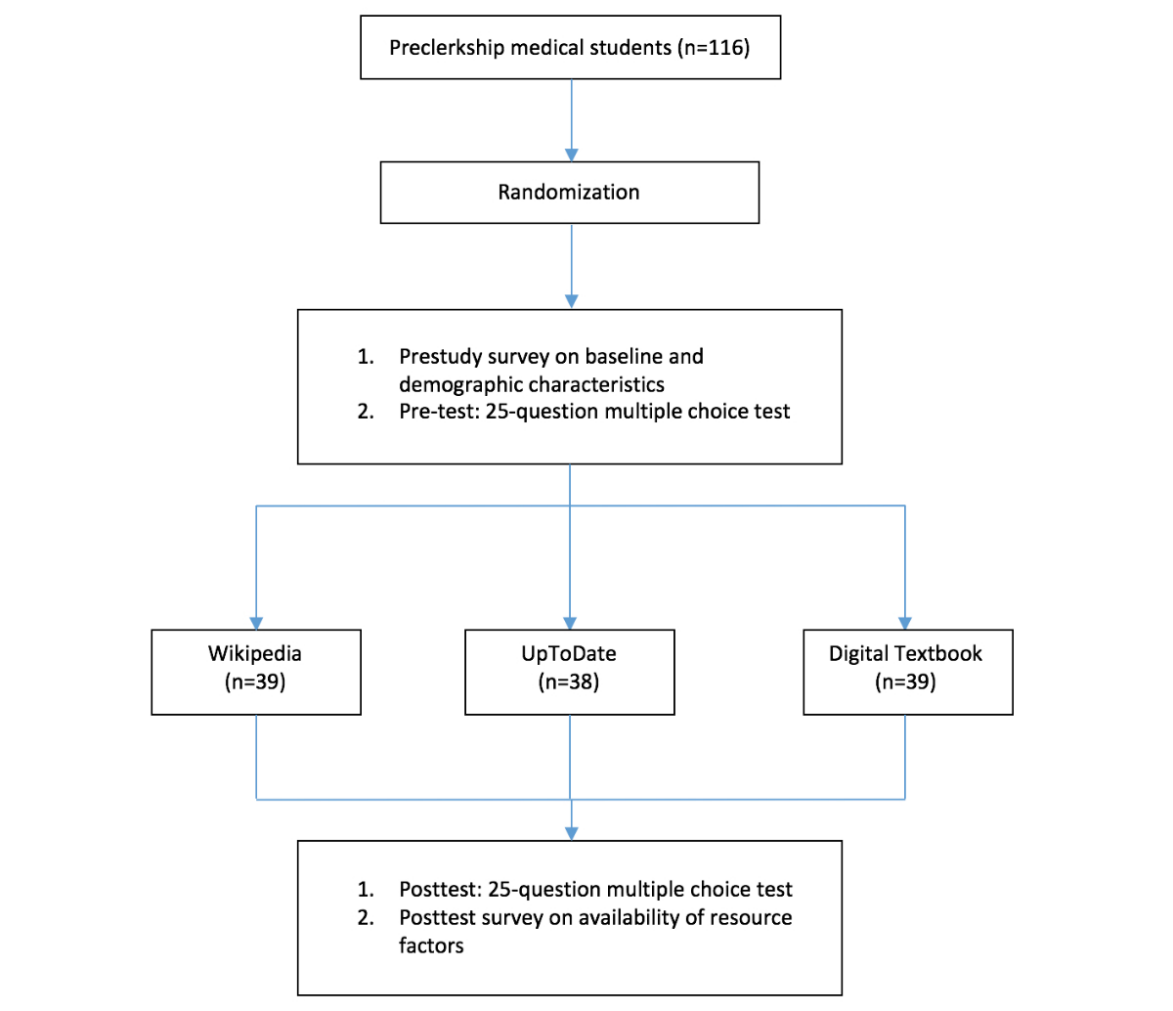
Study design.

The study methodology is summarized in Figure 1 **.** All participants completed a questionnaire of their demographics. Participants then had 30 min to complete a multiple-choice questionnaire (MCQ) pretest of 25 questions, wherein they could take written notes of questions they wanted to research further. After the pretest, participants had 30 min to research the questions using their assigned Web-based resources, during which they were allowed to create written notes. Finally, participants completed the same MCQ as a posttest, wherein they could use their written notes and general knowledge acquired from the intervention. They also completed a follow-up survey on the Web-based resources.

Data collection was done using two formats: in-person and online. Online administration was used to connect with remotely located participants and was conducted the same way as in-person collection. The two data collection methods differed only in degree of interactivity with the study coordinator. During in-person data collection, a study coordinator was present throughout the entire administration, who assigned participants to individual computers and only interacted with participants for consent, initial test setup, and notification of time remaining on each section. During online collection, screen-sharing software was used to track participant completion of the tests and to indicate time remaining on each section. In both scenarios, participants completed surveys, tests, and intervention using a standard Web browser and Google Forms software.

Study coordinators only answered questions regarding logistics (eg, remaining time) and did not advise participants on test content or Web-based resource navigation. Coordinators also ensured that participants only used their assigned intervention through direct observation or screen-sharing.

### Pretest Assessment

Participants completed a MCQ of 25 questions that emulated questions on the Medical Council of Canada Evaluation Exam (MCCEE). The MCCEE is a standardized examination administered by the Medical Council of Canada (Ottawa, Canada) to assess basic medical knowledge and readiness for postgraduate medical training in Canada. The content of the MCCEE is aimed toward graduating Canadian medical students to ensure that participants (ie, preclerkship medical students) would not have considerable prior knowledge. Test questions were retrieved from an MCCEE site, which is freely available online [20]. These questions were imported into a Google Forms questionnaire and delivered online. Questions were reviewed by 2 academic physicians (SG and JH) to ensure broad coverage of topics and appropriateness.

### Training Interventions

After recruitment, participants were randomized to one of three Web-based resources: (1) Wikipedia, (2) UpToDate, and (3) digital textbook. During the testing, participants each had 30 min to access the Web-based resource and could make notes using pencil and paper on any topics or questions on the test to research using the assigned intervention. Wikipedia and UpToDate were accessed using an Internet browser, with the participants logging into the latter using institutional accounts. The digital textbook, *Harrison’s Principles of Internal Medicine, 18th edition*, was accessed through institutional accounts.

Participants were limited to only their assigned interventions and were not allowed to search for additional information online. Moreover, participants were not provided with guidelines or strategies on how to access information. Coordinators tracked participant progress to ensure adherence with the assigned interventions. Within the allotted time, participants used a self-directed approach to research topics relevant to the MCQ.

### Posttest Assessment

To test for knowledge acquisition, participants completed the same MCQ administered at the beginning of the study. During this iteration, participants could refer to their written notes as a reference. After completing the test, participants answered a follow-up survey regarding five electronic resource factors: search functions, hyperlinks to other pages within the resource, references, open access editing, and expert editing. In the first section of the survey, participants rated the importance of each of the five factors in their learning. In the second section, participants then rated the availability of each of the factors within their assigned resource. For perceptions of importance, participants rated the five factors with respect to their general importance when using Web-based resources on a Likert-type scale of 1 to 5, where 1 represented *not important at all* and 5 represented *very important*. For perceptions of availability, participants rated the five factors with respect to only their assigned resource (Wikipedia, UpToDate, and digital textbook) on a Likert-type scale of 1 to 5, where 1 represented *not at all available* and 5 represented *very easy to access.*

### Outcome Measures

The primary outcome of the study was the difference in knowledge acquisition between the three groups as indicated by percentage scores on the MCQ. The tests were graded using a scoring key on a scale of 0 to 25. Each correct answer was awarded one point; incorrect answers or omissions were not penalized. Secondary outcome measures were the participants’ perceptions on availability of the five following factors: search functions, hyperlinks to other pages, references, open access editing, and expert editing.

### Sample Size

On the basis of previous research on knowledge acquisition using Web-based resources among medical trainees, 28 participants per group have been sufficient to detect significant differences between four groups [21]. To account for potential dropout, 116 participants were recruited.

### Statistical Analysis

Data were analyzed using Statistical Package for the Social Sciences (SPSS) version 20 (IBM Corp). Demographic variables were represented using descriptive statistics. All quantitative data were represented using means with standard deviations or medians with interquartile range, where appropriate. Categorical data were represented by count with frequency.

Primary analysis was intention-to-treat. To determine a difference in the MCQ scores across the three groups, a two-way mixed-factorial analysis of variance (ANOVA) was completed with one within-group factor (test: pretest and posttest) and one between-group factor (group: Wikipedia, UpToDate, and digital textbook). To determine whether there were any differences in participants’ perceptions of the importance and availability of the five resource factors for the resources (Wikipedia, UpToDate, and digital textbook), a Kruskal-Wallis test was used. Any significant effects on ANOVA or Kruskal-Wallis tests were further analyzed using Tukey honestly significant difference (HSD) and Mann Whitney *U* post hoc tests, respectively. Additionally, an exploratory analysis was conducted; two-way ANOVA was performed for posttest MCQ scores using highest level of education before medical school (group: masters, PhD, other professional degree) and assigned resource (group: Wikipedia, UpToDate, and digital textbook). The assumptions for the mixed ANOVA and two-way ANOVA were assessed and the appropriate corrections were applied for any violations [22]. Following statistical reporting recommendations, effect size was calculated using generalized eta squared (η_g_^2^) and Cohen measure (*d*) for ANOVA and Tukey HSD post hoc tests, respectively [23]. Alpha was set at .05 for all statistical tests.

## Results

### Demographics and Baseline Characteristics

A total of 116 preclerkship medical students were randomized and completed the study from April 2014 to December 2016. No participants were lost to follow-up. Participant demographics, prior resource use, and data collection format are provided in Table 1. Participants’ perceptions of the importance of several resource factors with respect to general Web-based resources are shown in Table 2. There were no significant differences between the groups on any of the five factors (*P*>.05).

### Primary Outcome

**Figure 2:**
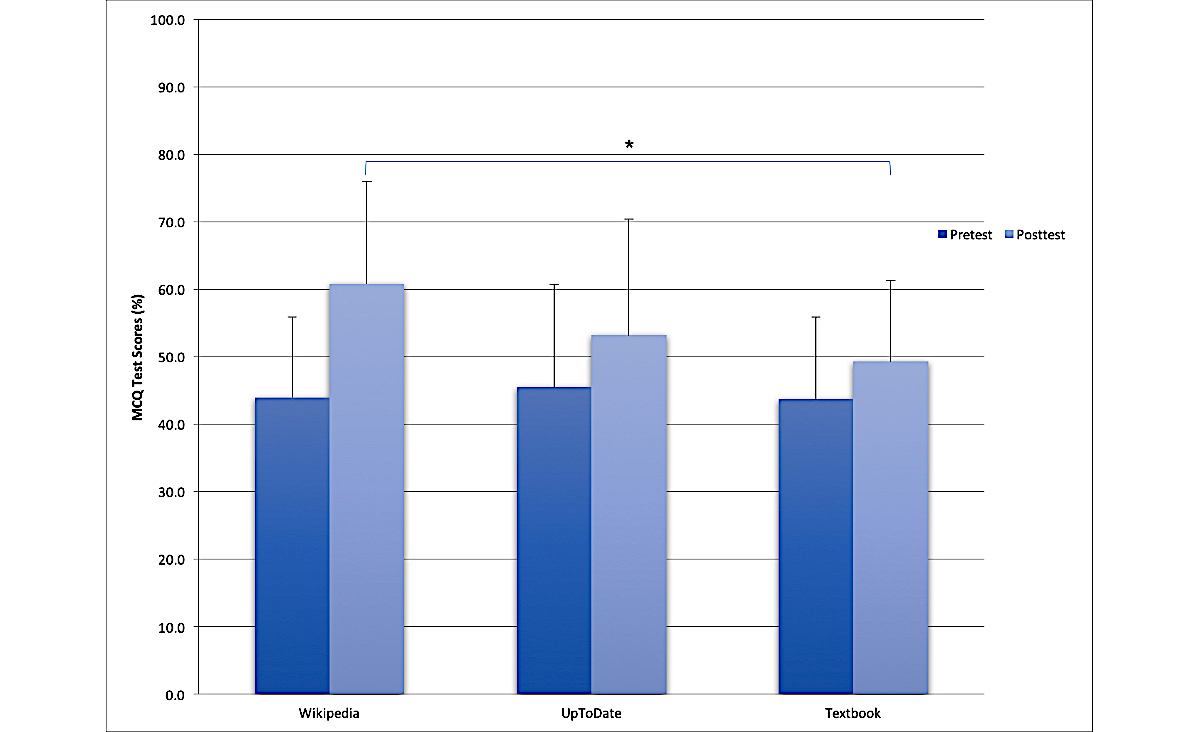
Bar graph of the mean percentage multiple-choice questionnaire (MCQ) test scores for the Wikipedia, UpToDate, and textbook groups at pretest and posttest. The bars indicate the standard deviation of the scores. Asterisks (*) indicate post hoc comparisons of *P*<.05.

MCQ responses for each group are shown in Figure 2. There were no significant differences between the three groups at pretest (Table 3). The ANOVA of the MCQ scores indicated a significant interaction between time and group effects (*F*_2,113_=10.07, *P*<.001, η_g_^2^=0.03). Post hoc analysis indicated that the Wikipedia group scored significantly higher on the posttest compared with the textbook (*P*=.01, *d*=0.86). There were no other significant post hoc pairwise comparisons between the other two groups. On the two-way ANOVA, there was no significant interaction between group assignment and highest education before medical school for posttest MCQ scores (*F*_4,106_=171.85, *P*=.51).

### Secondary Outcomes

Participants’ perceptions of the availability of resource factors within their assigned resource are shown in Table 4. There were significant differences between the groups on all the five factors (*P*<.001).

**Table 1:** Baseline demographic characteristics, prior resource use, and data collection format of participants.

Characteristic		Textbook group, (N=39)	UpToDate group, (N=38)	Wikipedia group, (N=39)
Sex, female, n (%)		16 (41)	16 (42)	8 (21)
Age (years), median (interquartile range)		23 (3)	23 (3)	23 (2)
**Highest level of training before medical school, n (%)**				
	Bachelor's degree	31 (80)	31 (82)	27 (69)
	Master’s degree	7 (18)	5 (13)	11 (28)
	PhD	1 (3)	1 (3)	1 (3)
	Other professional degree	0 (0)	1 (3)	0 (0)
**Used before study as learning resource, yes, n (%)**				
	Wikipedia	35 (90)	32 (84)	35 (90)
	UpToDate	29 (74)	28 (74)	29 (74)
	Digital textbooks	32 (82)	34 (90)	35 (90)
**Data collection format, n (%)**				
	In-person	26 (67)	24 (63)	24 (62)
	Online	13 (33)	14 (37)	15 (39)

**Table 2:** Participants’ perceptions of the importance of resource factors with respect to general Web-based resources in a poststudy survey. Values are median ratings with interquartile range in parentheses, where 1 is *not important at all* and 5 is *most important*.

Resource factor	Wikipedia group, median (IQR^a^)	UpToDate group, median (IQR)	Textbook group, median (IQR)	*P* value
Search function	5.0 (1.0)	5.0 (0)	5.0 (0)	.06
Hyperlinks	4.0 (2.0)	4.0 (2.0)	4.0 (2.0)	.42
References	4.0 (1.0)	3.0 (2.0)	4.0 (2.0)	.44
Open access editing	2.0 (2.0)	2.0 (2.0)	2.0 (2.0)	.18
Expert editing	4.0 (1.0)	4.0 (1.0)	4.0 (1.0)	.82

^a^IQR: interquartile range.

**Table 3:** Multiple-choice questionnaire results for all three groups.

MCQ^a^score	Wikipedia group, mean % (SD)	UpToDate group, mean % (SD)	Textbook group, mean % (SD)	*P* value		
				Wikipedia-UpToDate	Wikipedia-textbook	UpToDate-textbook
Pretest	44.10 (11.70)	45.46 (15.43)	43.90 (12.26)	.65	.95	.60
Posttest	61.03 (15.29)	55.26 (15.31)	49.23 (11.94)	.08	<.001^b^	.07

^a^MCQ: multiple-choice questionnaire.

^b^Indicates statistically significant findings among pairwise comparisons (*P*<.05).

**Table 4:** Participants’ perceptions of five resource factors with respect to their assigned resource (Wikipedia, UpToDate, and textbook) in a poststudy survey. Values are median ratings with interquartile range in parentheses, where 1 is *not at all available* and 5 is *very easy to access*.

Resource factor	Wikipedia group, median (IQR^a^)	UpToDate group, median (IQR)	Textbook group, median (IQR)	*P* value		
				Wikipedia-UpToDate	Wikipedia-textbook	UpToDate-textbook
Search function	5.0 (1.0)	4.0 (2.0)	3.0 (2.0)	.25	<.001	<.001
Hyperlinks	4.0 (1.0)	4.0 (2.0)	2.0 (2.0)	>.99	<.001	<.001
References	4.0 (1.0)	5.0 (1.0)	4.0 (1.0)	<.001	>.99	<.001
Open access editing	4.0 (2.0)	1.0 (2.0)	1.0 (0)	<.001	<.001	.63
Expert editing	3.0 (2.0)	4.5 (2.0)	4.0 (2.0)	<.001	<.001	.69

^a^IQR: interquartile range.

## Discussion

This study demonstrates that Wikipedia can be effectively used as a resource for short-term knowledge acquisition by medical students. Specifically, the Wikipedia group had significantly better posttest performance on an MCQ examination based on the MCCEE compared with the digital textbook group. Additionally, the Wikipedia group trended toward better posttest performance compared with the UpToDate group. Finally, the UpToDate group trended toward better posttest performance compared with the digital textbook group. These latter two comparisons, however, were not significant. This is the first trial directly evaluating the impact of Wikipedia on medical knowledge acquisition beginning to address a gap identified in a recent Cochrane Review [24].

These results may be explained by differences between the three resources with respect to the availability of certain resource functions and familiarity. First, Wikipedia’s search functions and hyperlinks were rated significantly higher than the digital textbook (these factors were not significantly different between Wikipedia and UpToDate), suggesting that participants were able to find information more easily. In addition, more participants reported using Wikipedia as a learning resource at baseline compared with UpToDate and digital textbook. Increased familiarity with Wikipedia is supported by literature, underscoring the high prevalence of its use among medical students [3,9].

Ease of navigation, afforded by better search functions and hyperlinks and familiarity, may have placed a lower cognitive load on students using Wikipedia compared with a digital textbook. According to cognitive load theory, there are limitations or loads on the amount of novel information that the brain can process [25]. We hypothesize that a lower cognitive load allowed students to more efficiently access and acquire knowledge. Our interpretation is commensurate with previous work exploring mental exertion in medical students. Using eye metrics such as task-evoked pupillary response and eye fixation, one group found that UpToDate was associated with higher levels of mental exertion compared with Wikipedia [26].

These findings suggest a potential role for Wikipedia in medical education. However, Wikipedia use is currently discouraged in the academic community because of concerns regarding its accuracy and reliability [11,12,15]. Additionally, participants in our study held negative attitudes toward Wikipedia, as they perceived it as having fewer references and less expert editing compared with UpToDate and a digital textbook.

Whereas some criticism is warranted, there is strong evidence supporting the use of Wikipedia in health care. A recent systematic review of Wikipedia found more studies reporting positive than negative evaluations of article quality [27]. Wikipedia has also been endorsed in patient and nursing education because of the reliability and accuracy of its health-related articles [17,18,28]. Furthermore, the claim that Wikipedia lacks sufficient editorial controls is tenuous, as it has its own editorial mechanisms. WikiProject Medicine, a user group founded in 2004, is a distributed expert review board dedicated to coordinating medical content on Wikipedia. They also publish a style manual with recommendations on how to write health-related articles and grade articles per quality measures [29]. Finally, Wikipedia offers an advantage that subscription-based resources cannot—free access. This feature makes it available to medical students who may not have subscriptions.

There are several strengths of this study. First, there was excellent integrity of study participation and data, as there was no participant dropout and no missing data. Second, the generalizability of the findings benefit from the inclusion of students from multiple medical schools. Finally, this is the first known study that investigated the impact of Wikipedia as an electronic resource using an RCT design.

Our findings must be framed within the context of the study limitations. First, participants who did not finish the pretest within 30 min would not have known which topics to search to answer missed questions. Second, posttest scores may have been inflated, as the participants who correctly answered select questions in the pretest would have had more time to answer the remaining questions. These two limitations, however, would have been uniform across the three groups, thereby, likely not having contributed to observed differences between the groups. Third, participants may only have enrolled in the study if they had experience in using electronic resources, which could have introduced selection bias. Although this bias could impair the generalizability of the findings, its impact is likely minimal, as there is evidence that up to 94% of medical students use Wikipedia [9] **.** In addition, the nonblinded nature of our study may have impacted study results. It is, however, not possible to conduct a truly blinded randomized trial for many educational interventions. Fourth, Wikipedia and UpToDate are dynamic resources that are edited as medical knowledge evolves. The replicability of this study may be compromised with time as the health-related entries on these dynamic resources change. Finally, our study was potentially underpowered as the Wikipedia group trended toward but did not have significantly better knowledge acquisition compared with the UpToDate group. As this is the first study of its kind, it is possible that our sample size calculation was inaccurate because of a dearth of appropriate comparative literature.

Although this study and others suggest there is educational value in Wikipedia, few medical schools have seriously explored its potential as a knowledge acquisition resource. This stance may be shortsighted, as many trainees use this resource and will likely continue as practicing physicians [5,10]. Medical schools may benefit from considering the use of Wikipedia in their curricula, such as enlisting students to create and edit medically focused articles. A recent study found that medical students who edited Wikipedia for course credit not only improved the quality of the articles but also enjoyed the editing experience [30]. Social-constructivist learning models theorize that participation in content development allows learners to become better acquainted with knowledge as active agents of learning [31]. Using this theoretical approach, future research could explore how trainee involvement with the creation and development of content on Wikipedia relates to their learning and knowledge acquisition.

## Abbreviations

ANOVA: analysis of variance
HSD: honestly significant difference
IQR: interquartile range
MCQ: multiple-choice questionnaire
MCCEE: Medical Council of Canada Evaluation Exam
RCT: randomized controlled trial
SD: standard deviation

## Appendix

Multimedia Appendix 1 CONSORT‐EHEALTH checklist (V.1.6.1).
